# Supplementary material for: Dynamic chloride ion adsorption on single iridium atom boosts seawater oxidation catalysis
Source: Nat Commun. 2024 Mar 4;15:1973. doi: 10.1038/s41467-024-46140-y (PMC10912682; doi:10.1038/s41467-024-46140-y)
Supplement: Supplementary file 1 — Supplementary Information [file 41467_2024_46140_MOESM1_ESM.pdf]

This PDF file includes:

Supplementary Fig. 1 to 56

Supplementary Table 1 to 7

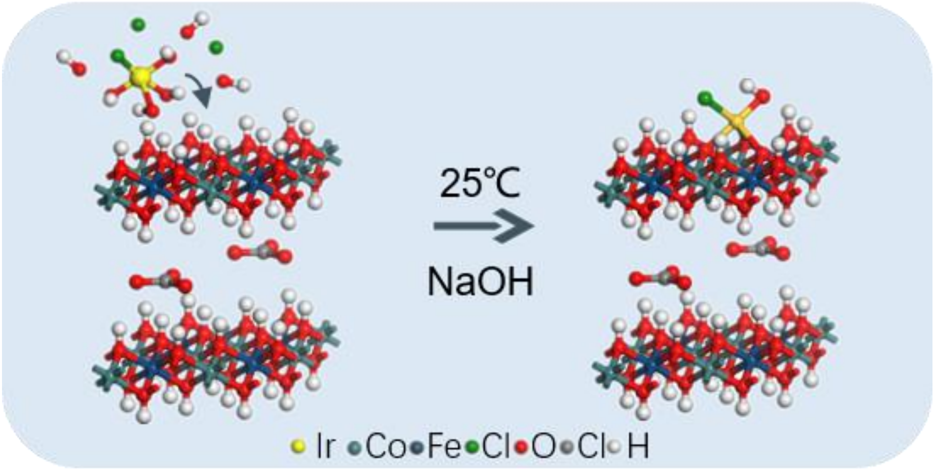

Supplementary Fig. 1 Schematic illustration of the preparation process of Ir/CoFe-LDH.

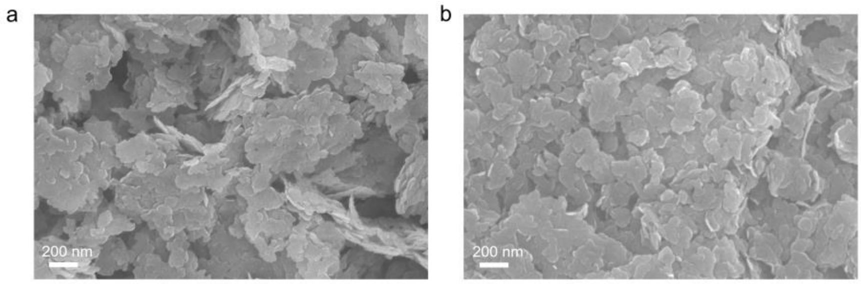

Supplementary Fig. 2 High-magnification SEM images of (a) CoFe-LDH and (b) Ir/CoFe-LDH before OER test.

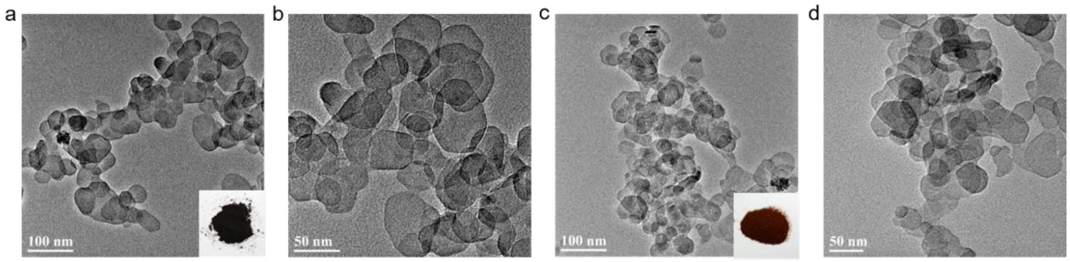

Supplementary Fig. 3 TEM images of (a) & (b) Ir/CoFe-LDH, and (c) & (d) CoFe-LDH. Digital photographs inserted in (a) and (c) show that the color of the sample gets darker after loading Ir.

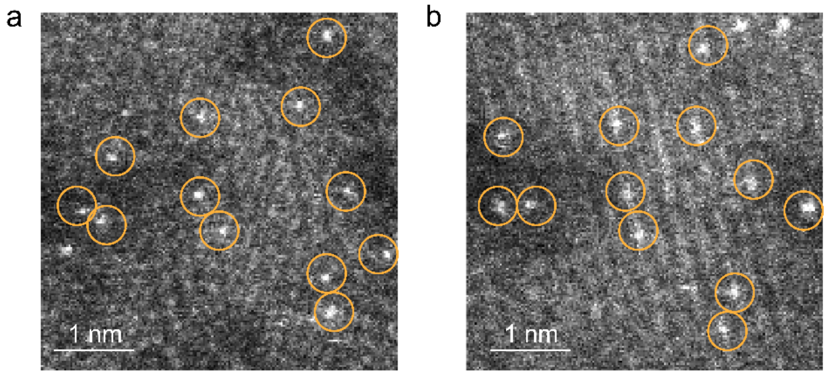

Supplementary Fig. 4 HAADF-STEM image of Ir/CoFe-LDH (a) without and (b) with a 30° tilt. The isolated Ir atoms are highlighted with yellow circles.

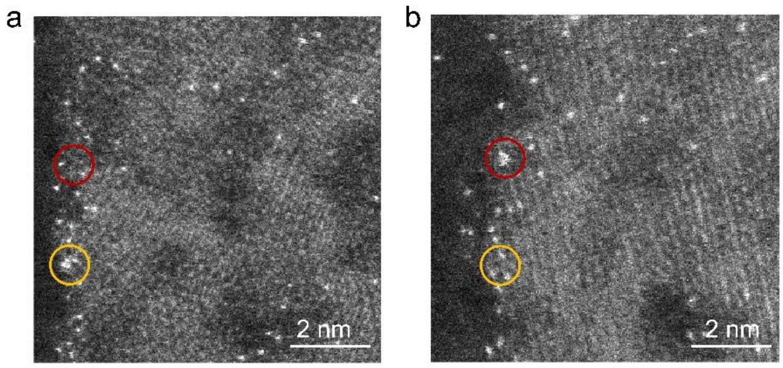

Supplementary Fig. 5 HAADF-STEM image of Ir/CoFe-LDH (a) without and (b) with a 30° tilt. The corresponding Ir atoms are marked with yellow and red circles, respectively, suggesting that the visual aggregation of Ir atoms may be caused by viewing angles.

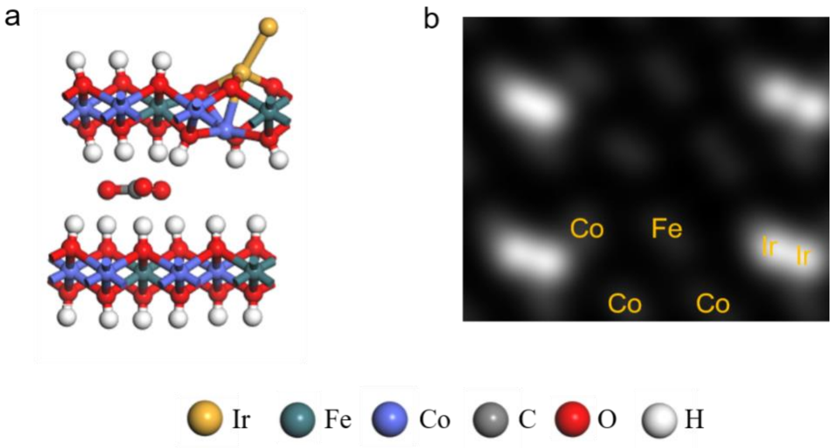

Supplementary Fig. 6 (a) Schematic diagram depicting the atomic model of an Ir-Ir axial dimer from DFT simulation and (b) the corresponding Dr. probe simulation.

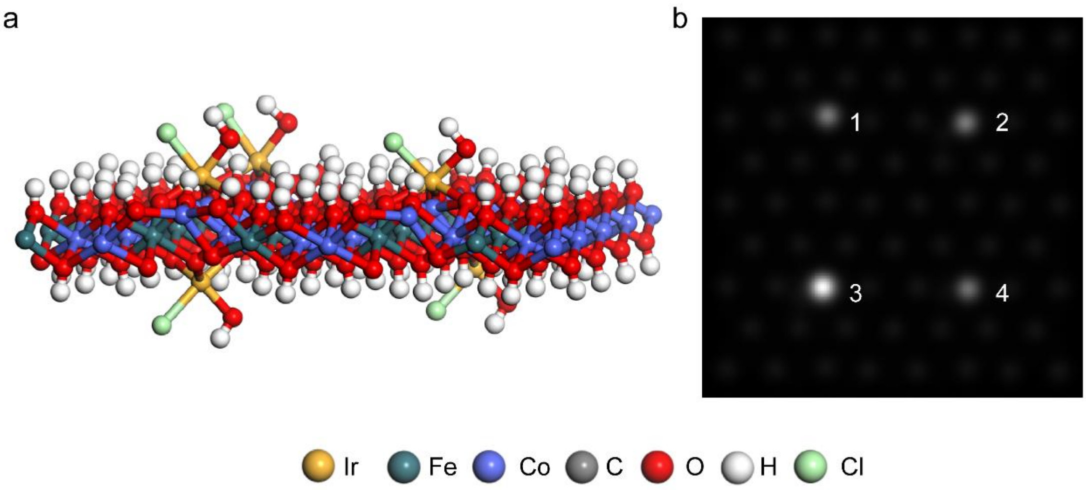

Supplementary Fig. 7 (a) Schematic diagram showing the atomic model of single Ir atom on CoFe-LDH and (b) the corresponding Dr. probe simulation.

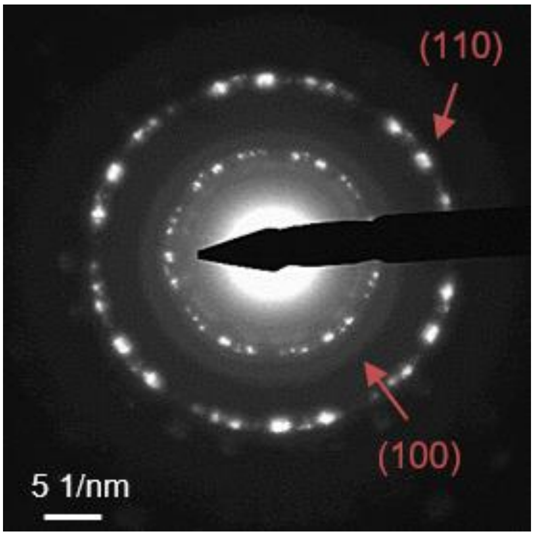

Supplementary Fig. 8 The SAED pattern of Ir/CoFe-LDH.

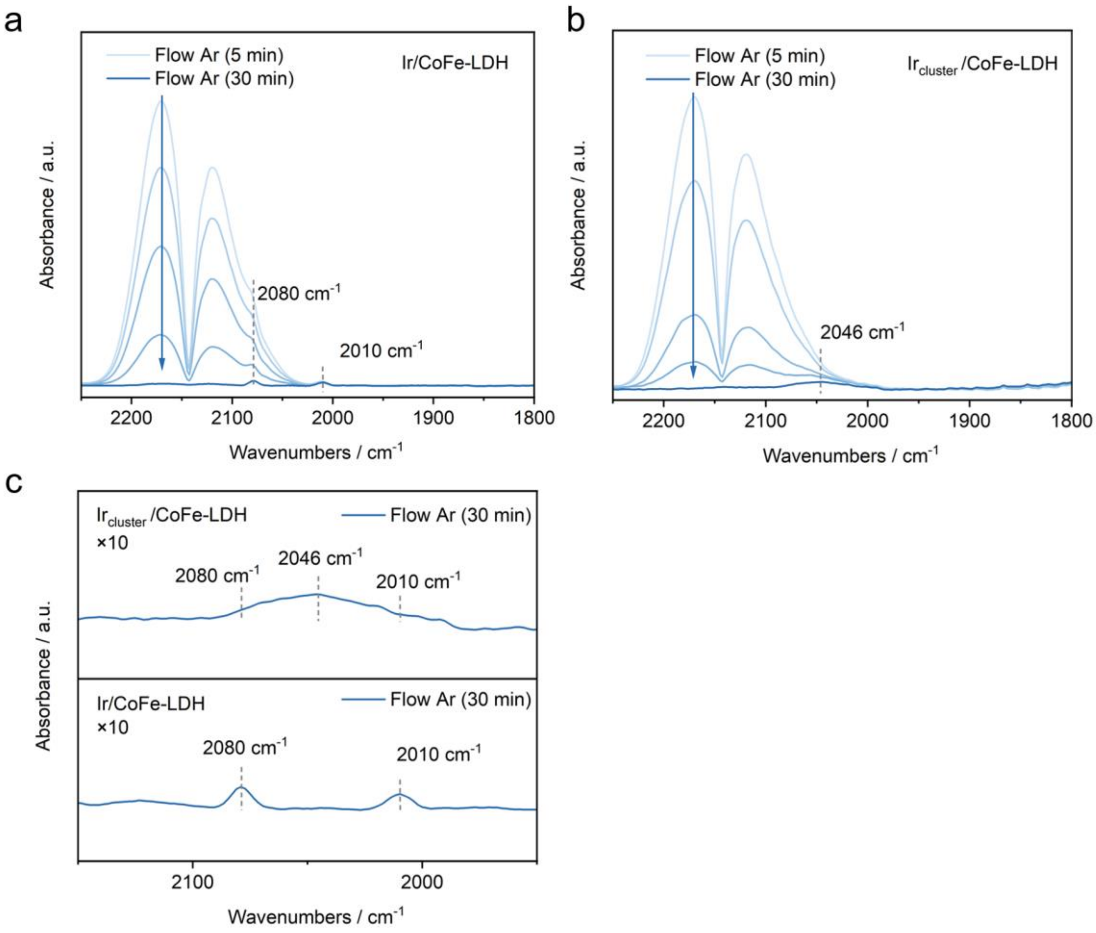

Supplementary Fig. 9 DRIFTS spectra of the CO peak on (a) Ir/CoFe-LDH (b) $Ir_{cluster}$ /CoFe-LDH during Ar purging. (c) DRIFTS spectra of chemically adsorbed CO over Ir/CoFe-LDH (bottom) and $Ir_{cluster}$ /CoFe-LDH (top) samples.

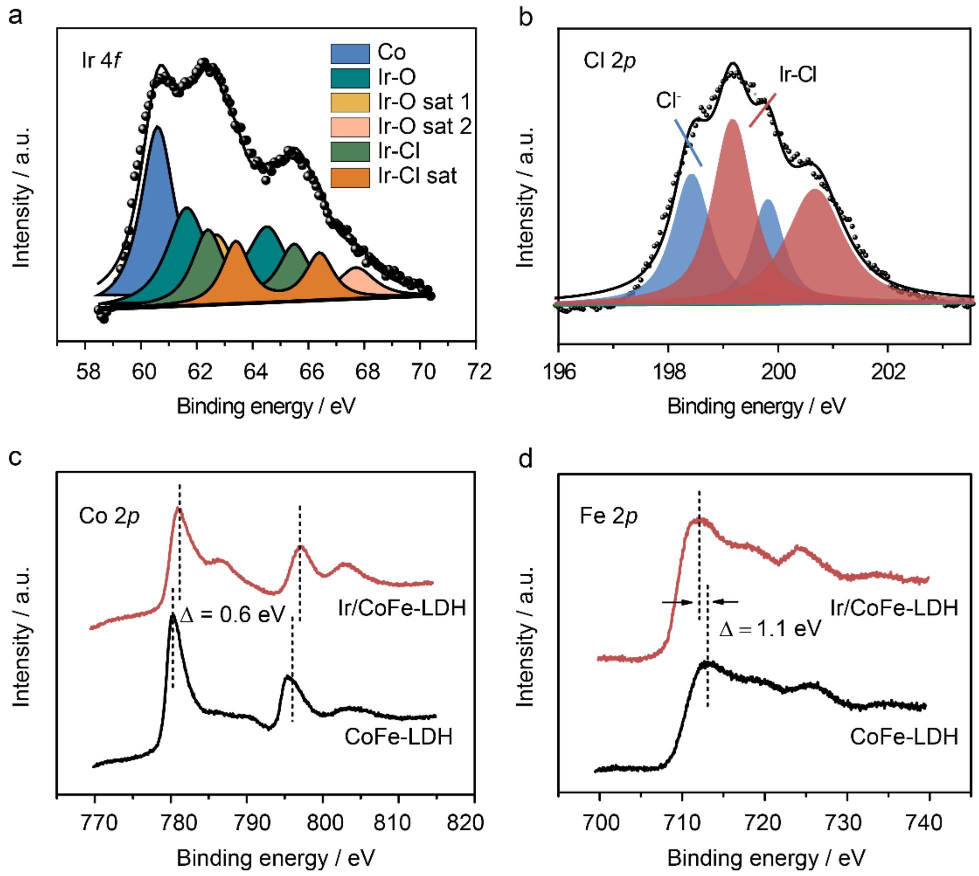

Supplementary Fig. 10 The high-resolution XPS spectra of (a) Ir, (b) Cl, (c) Co, and (d) Fe for CoFe-LDH and Ir/CoFe-LDH.

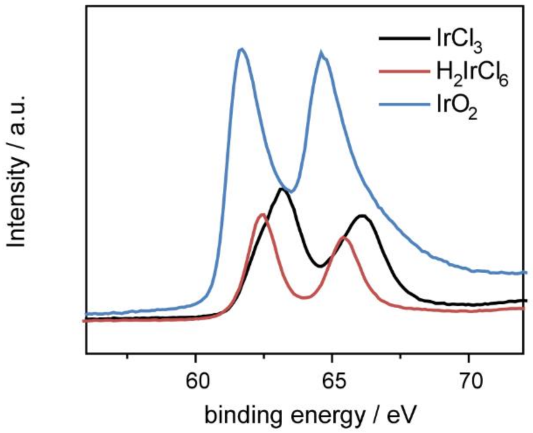

Supplementary Fig. 11 High-resolution XPS spectra of $IrCl_3$, H_2IrCl_6 and IrO_2.

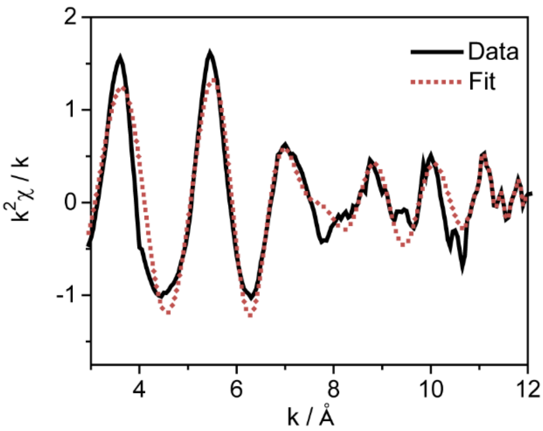

Supplementary Fig. 12 The EXAFS k space fitting curve of Ir/CoFe-LDH.

Supplementary Table 1 EXAFS data of Ir/CoFe-LDH.

**Sample**	**Shell**	**CN**	**R (Å)**	**$\Delta\sigma^2\times10^3$ (Å^2)**	**ΔE_0 (eV)**
Ir/CoFe-LDH	Ir-O	3.1	1.99	0.006	5.60
	Ir-Cl	2.5	2.33	0.004	9.66
	Ir-(O)-Co	2.0	3.20	0.005	12.50

CN: coordination number, *R*: distance between absorber and backscatterer atoms, $\Delta\sigma^2$: disorder term, ΔE_0: inner potential correction. Error bars are estimated to be CN: ± 15%, *R*: ± 0.02 Å, $\Delta\sigma^2$: ± 20%, and ΔE_0: ± 20%.

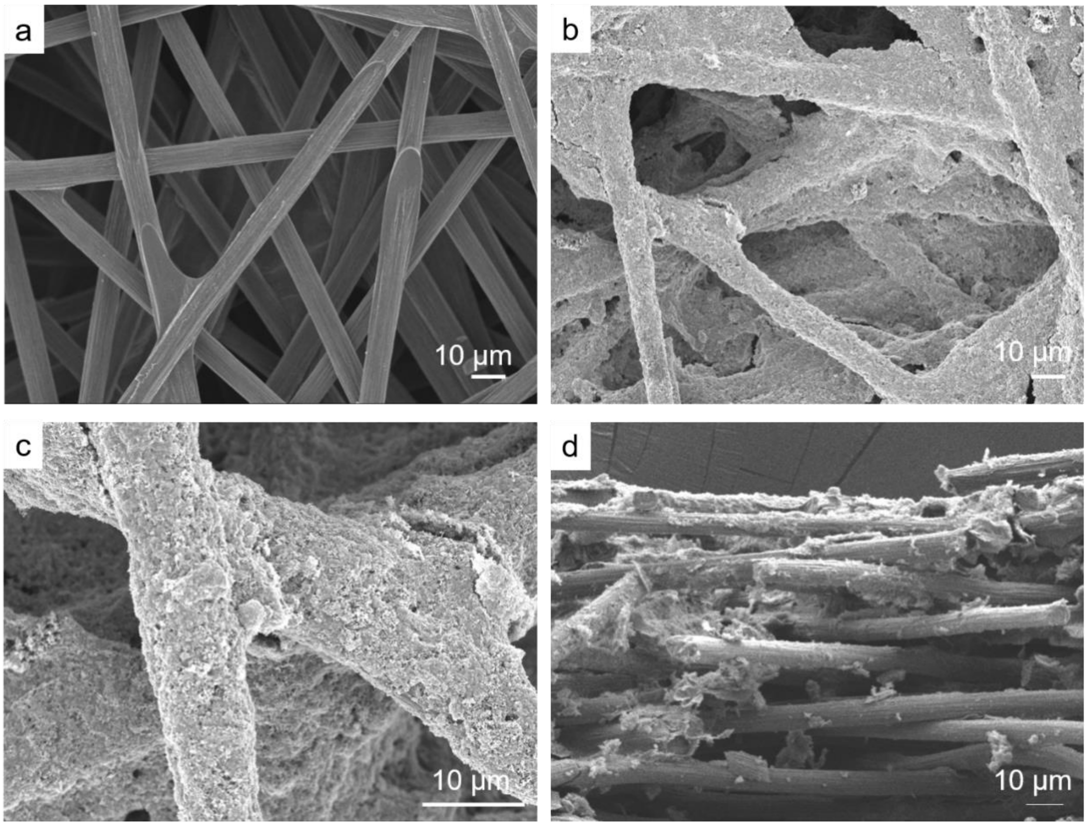

Supplementary Fig. 13 SEM images of (a) Carbon fiber paper, (b-c) Ir/CoFe-LDH loaded on carbon fiber paper under different magnifications. (d) cross-section view of Carbon fiber paper loaded with catalysts.

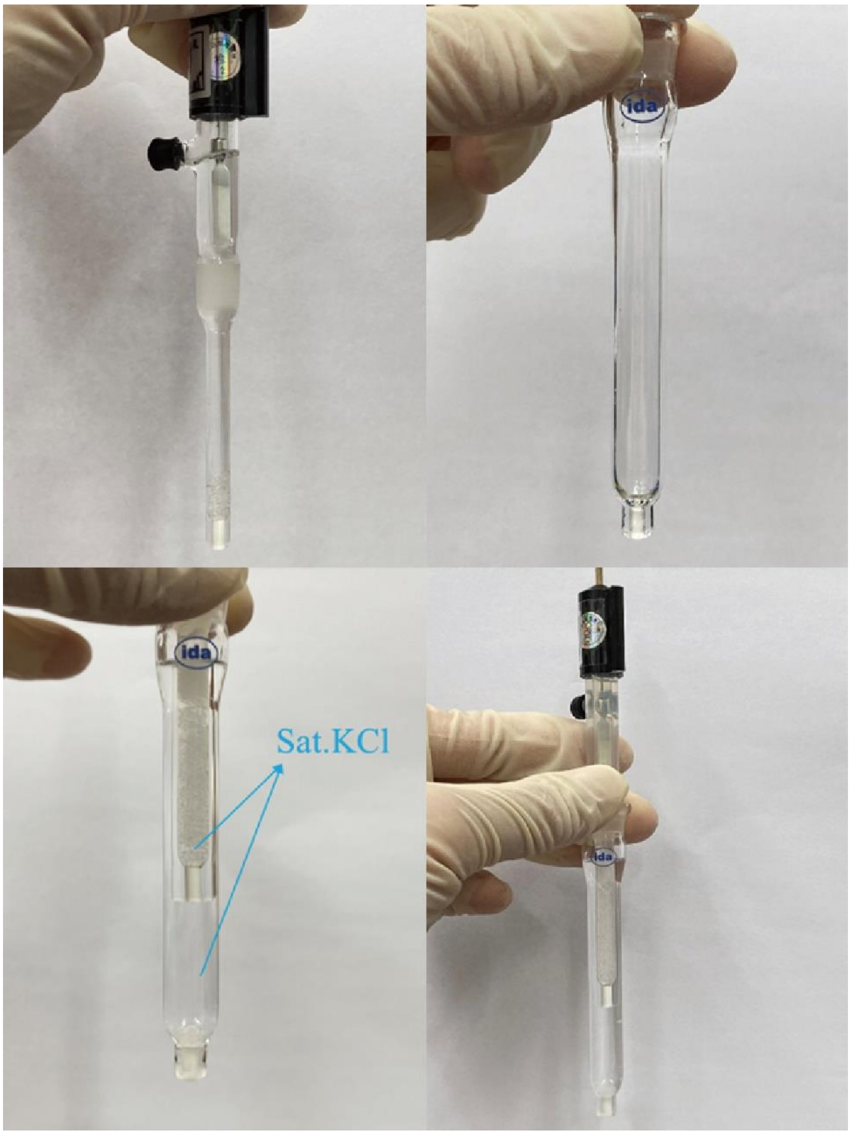

Supplementary Fig. 14 Digital photographs of SCE reference electrode with salt bridge.

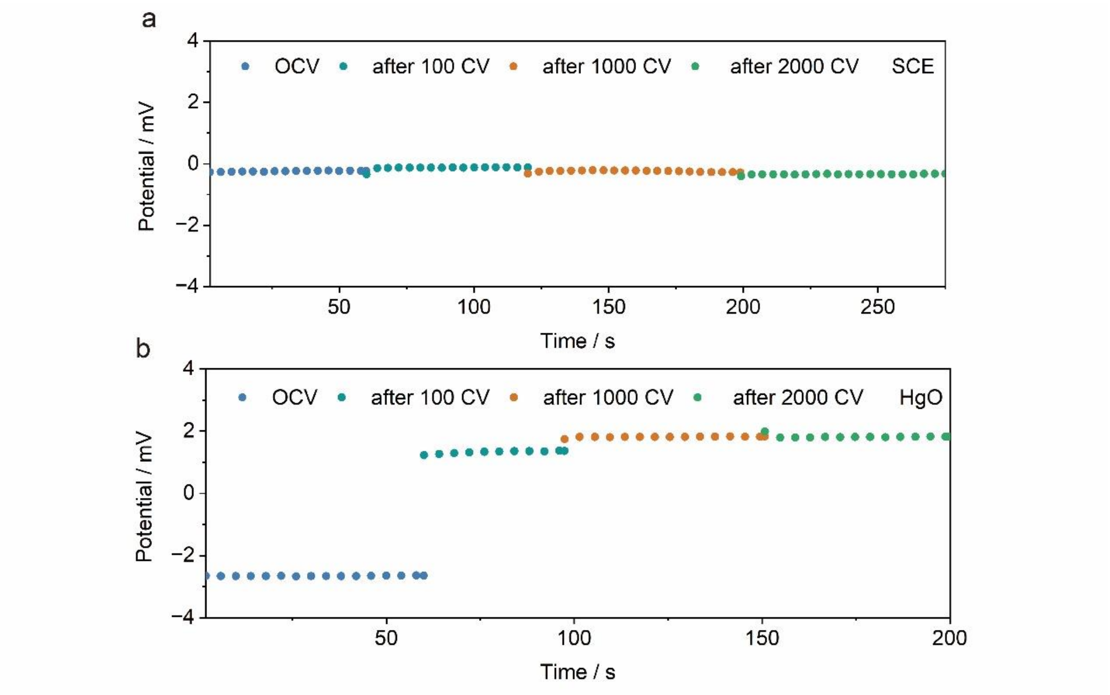

Supplementary Fig. 15 Using (a) SCE and (b) HgO as reference electrodes (with double salt bridge), OCV curves were obtained after conducting 100 CV, 1,000 CV, and 2,000 CV tests in 6 M NaOH + 2.8 M NaCl.

Supplementary Note. 1 The SCE double salt bridge, which was purchased from Tianjin Aida Hengsheng Technology Development Co., Ltd (as shown in supplementary Fig. 14), consists of saturated KCl. In the choice of electrolyte for testing, the use of 6 M NaOH mirrors the alkalinity levels typically employed in industrial alkaline water electrolysis. Moreover, according to our previous work[1], it is notable that the maximal solubility of NaCl in 6 M NaOH is 2.8 M, as excess NaCl concentration beyond this threshold results in crystalline precipitation. Importantly, in practical scenarios, electrolyte salt concentrations are unlikely to exceed this limit. By conducting tests under these conditions, we aim to showcase the electrode's performance at its utmost capacity.

We conducted prolonged cyclic tests on the saturated calomel electrode (SCE) double salt bridge in a 6 M NaOH + 2.8 M NaCl environment (as shown in supplementary Fig. 15), unequivocally validating the robustness of SCE under conditions of elevated alkalinity and high salt concentration. We employed Ir/CoFe-LDH as the working

electrode, Pt as the counter electrode, and utilized both the SCE double salt bridge and HgO double salt bridge as reference electrodes. CV measurements were performed in the range of 0.146 V to 1.146 V vs. HgO and 0 V to 1 V vs. SCE, at a scan rate of 50 mV/s in the 6 M NaOH + 2.8 M NaCl solution. Prior to the CV measurements, the SCE and HgO reference electrodes were calibrated against standard SCE and standard HgO electrodes, respectively. In order to assess the extent of potential deviation under demanding operating conditions, the OCV calibration was conducted at different stages, namely after 100 cycles (4,000 s), after 1,000 cycles (12 h), and after 2,000 cycles (24 h). The results indicate that under prolonged continuous operation (after 2,000 cycles, 24 h), the SCE exhibited fluctuations within 0.2 mV (as shown in supplementary Fig. 15), while the HgO displayed fluctuations within 4 mV. This finding confirms the stability of SCE under harsh conditions. Routine testing typically lasted only for a few minutes and is far less demanding compared to these stringent conditions. Therefore, by regularly replacing the solution in the salt bridge, it is possible to maintain the long-term operation of the reference electrode.

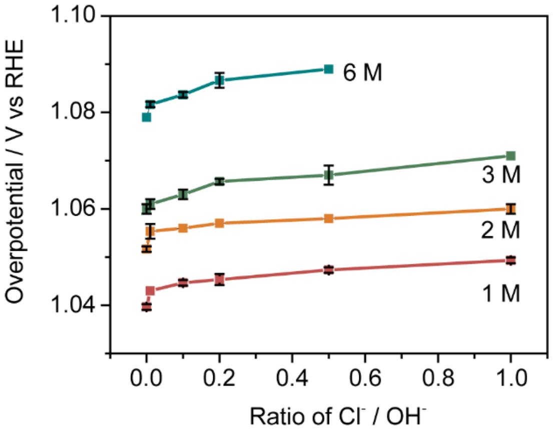

Supplementary Fig. 16 The RHE conversion in 1 M, 2 M, 3 M, and 6 M NaOH with different NaCl: NaOH ratios.

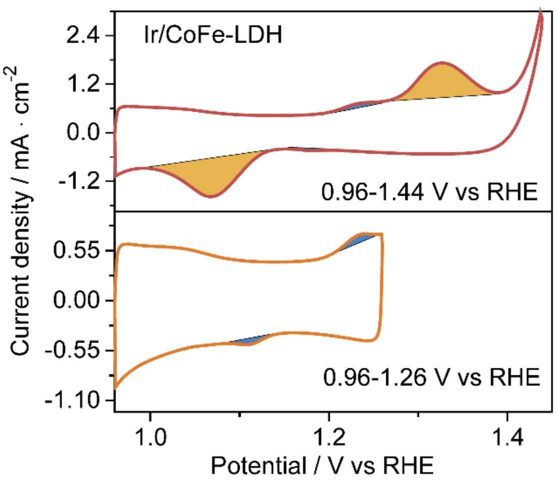

Supplementary Fig. 17 CV curves of Ir/CoFe-LDH recorded in NaOH under selected potential.

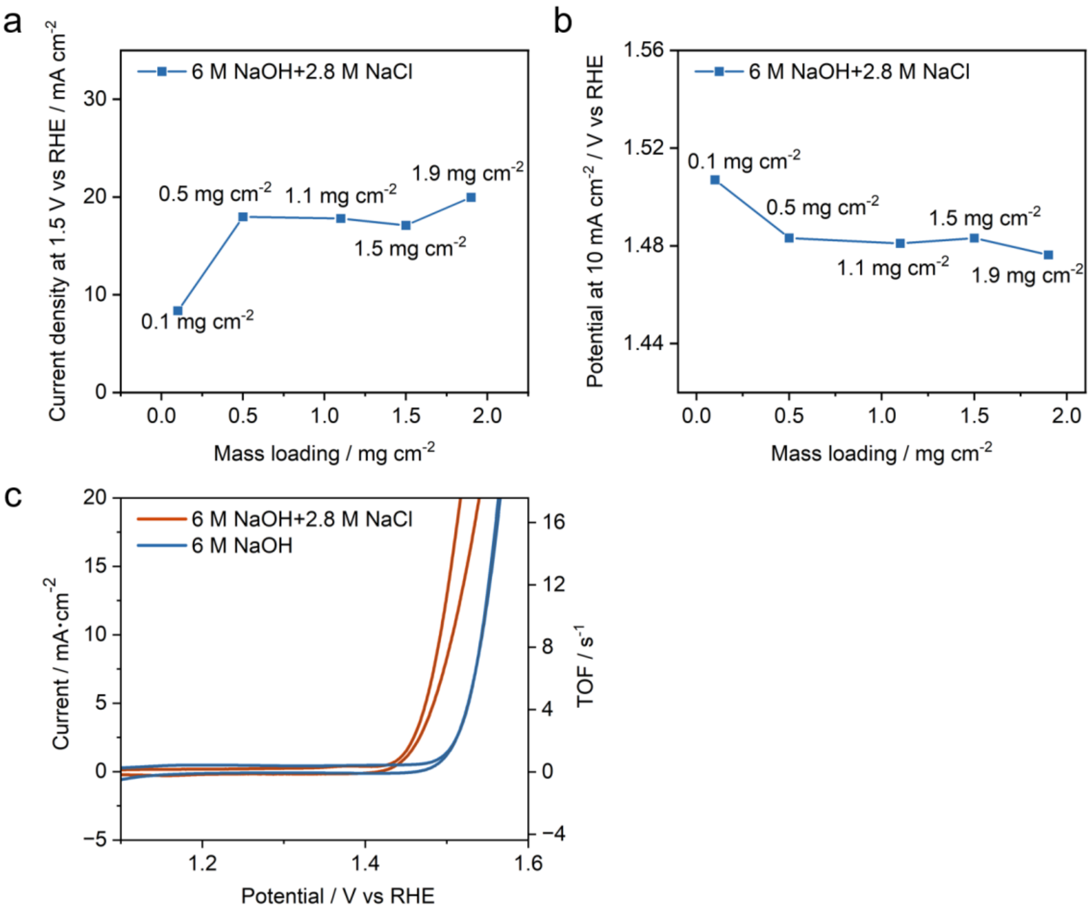

Supplementary Fig. 18 (a) Correlation between Ir/CoFe-LDH mass loading and

potential at a current density of 10 mA/cm², (b) correlation between Ir/CoFe-LDH mass loading and current density at 1.5 V vs. RHE, and (c) comparison of CV curves and TOF of Ir/CoFe-LDH (0.1 mg/cm^2 mass loading) recorded in 6 M NaOH and 6 M NaOH + 2.8 M NaCl.

Supplementary Note 2. The turnover frequency (TOF) per Ir site was calculated according to the following equation:

$$\text{TOF }(\text{O}_2/\text{s}) = \frac{\text{total oxygen turnover per geometric area}}{\text{number of active Ir sites per geometric area}}$$

The total number of oxygen turnover could be obtained by current density using the following formula:

Total number of oxygen turnover:

$$= (|j|\text{ mA cm}^{-2})\left(\frac{1\text{C}}{1{,}000\text{ mA}\cdot\text{s}}\right)\left(\frac{1\text{ mol e}^-}{96{,}485.3\text{ C}}\right)\left(\frac{1\text{ mol O}_2}{4\text{ mol e}^-}\right)\left(\frac{6.022\times10^{23}\text{ molecules O}_2}{1\text{ mol O}_2}\right)$$

$$=1.560\times10^{15}\ |j|\text{ cm}^{-2}\text{s}^{-1}$$

The number of Ir active sites in Ir/CoFe-LDH was calculated according to the mass loading of Ir on the electrode (dispersed Ir atoms were anchored on the surface of CoFe-LDH, which acted as the active sites to catalyze the oxygen evolution reaction):

Number of Ir active sites:

$$= \left(\frac{\text{catalyst loading per geometric area}\times\text{Ir wt.\%}}{\text{Ir Mw}}\right)\times\left(\frac{6.022\times10^{23}\text{ Ir atoms}}{1\text{ mol Ir}}\right)$$

$$= \left(\frac{0.1\text{ mg cm}^{-2}\times0.57\text{ wt.\%}}{192.217\text{ g mol}^{-1}}\right)\times\left(\frac{6.022\times10^{23}\text{ Ir atoms}}{1\text{ mol Ir}}\right) = 1.785\times10^{15}\text{cm}^{-2}$$

So, TOF can be calculated:

$$\text{TOF} = \frac{1.560\times10^{15}\text{ cm}^{-2}\text{s}^{-1}}{1.785\times10^{15}\text{ cm}^{-2}}\times|j| = 0.874\times|j|\ \text{s}^{-1}$$

where J is the current density (mA cm^{-2}). At a current density of 10 mA cm^{-2}, the TOF is 8.74 s^{-1} (Supplementary Fig. 18c).

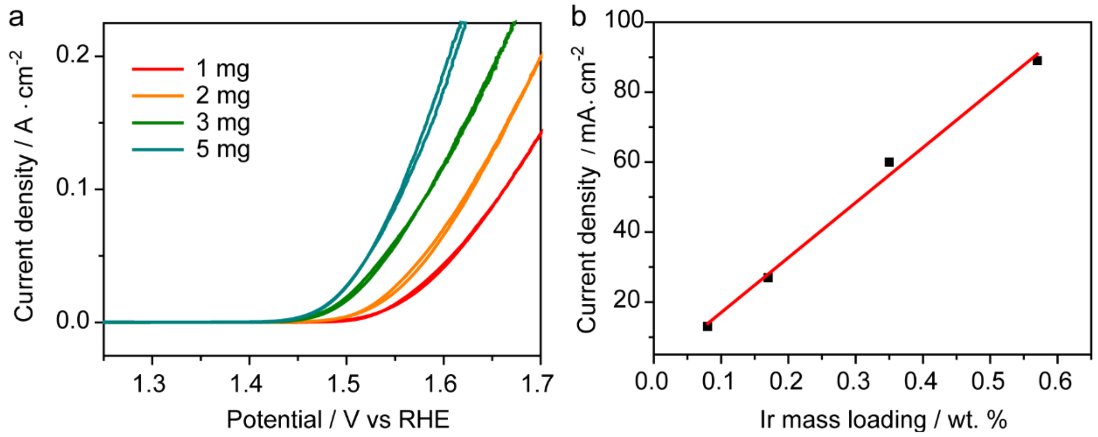

Supplementary Fig. 19 (a) The CV curves of Ir/CoFe-LDH prepared by adding different amounts of $IrCl_3$ to the CoFe-LDH colloid suspension recorded in 1 M NaOH + 0.5 M NaCl and (b) the relationship between Ir loading amount and current density at 1.55 V vs. RHE.

Supplementary Table 2. The relationship between Ir loading amount and OER current density at 1.55 V vs. RHE.

Sample name	Added $IrCl_3$ (mg)	Actual Ir mass loading (wt.%)	Current density at 1.55 V vs. RHE (mA cm^{-2})
1	1	0.08	13.7
2	2	0.17	27.4
3	3	0.35	60.0
4	5	0.57	89.7

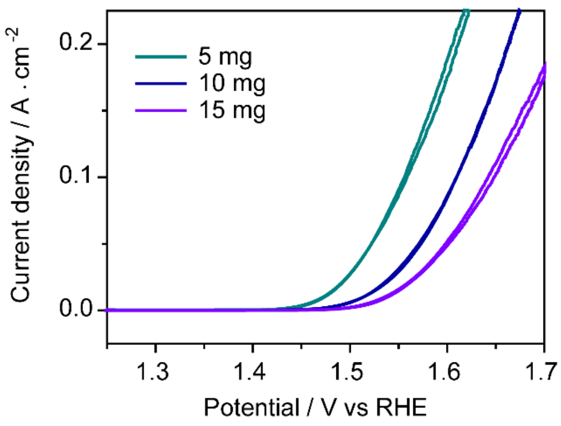

Supplementary Fig. 20 The CV curves of Ir/CoFe-LDH prepared by adding different amounts (more than the optimal quantity) of $IrCl_3$ to the CoFe-LDH colloid suspension recorded in 1 M NaOH + 0.5 M NaCl.

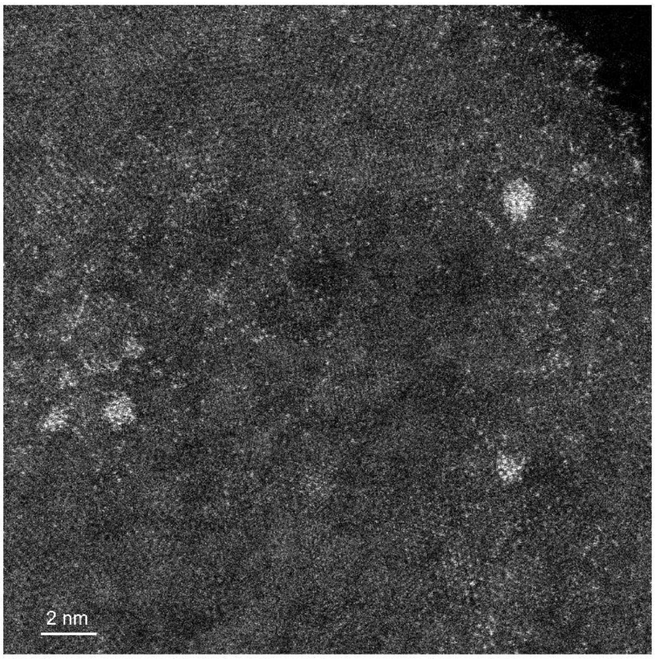

Supplementary Fig. 21 HAADF-STEM image of Ir/CoFe-LDH prepared by adding 10 mg of $IrCl_3$, which is much higher than the maximum amount to achieve Single-Atom catalyst.

Supplementary Table 3. The relationship between Ir loading amount and OER current density at 1.55 V vs. RHE.

Sample name	Added $IrCl_3$ (mg)	Actual Ir mass loading (wt.%)	Current density at 1.55 V vs. RHE (mA cm^{-2})
1	5	0.57	89.7
2	10	0.84	32.6
3	15	1.18	15.0

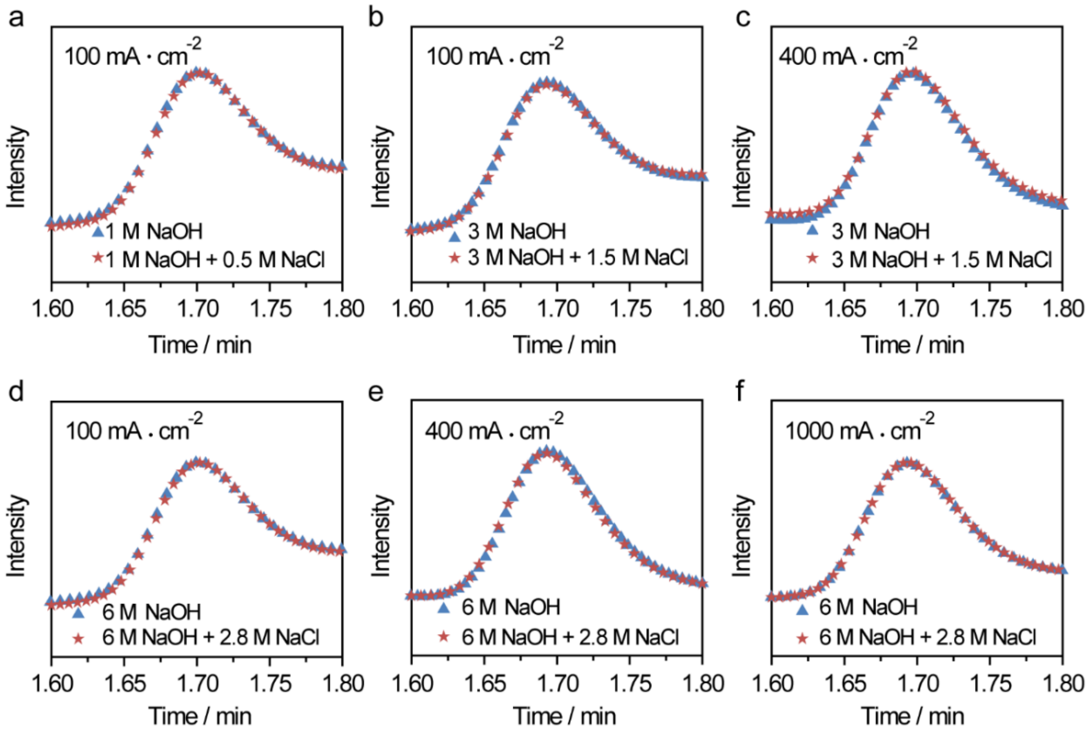

Supplementary Fig. 22 Gas chromatography O_2 signal collected from the electrolyzer operating at (a) 100 mA/cm^2 in 1 M NaOH and 1 M NaOH + 0.5 M NaCl; (b) 100 mA/cm^2 in 3 M NaOH and 3 M NaOH + 1.5M NaCl; (c) 400 mA/cm^2 in 3 M NaOH and 3 M NaOH + 1.5 M NaCl; (d) 100 mA/cm^2 in 6 M NaOH and 6 M NaOH + 2.8 M NaCl, (e) 400 mA/cm^2 in 6 M NaOH and 6 M NaOH + 2.8 M NaCl; and (f) 1,000 mA/cm^2 in 6 M NaOH and 6 M NaOH + 2.8 M NaCl.

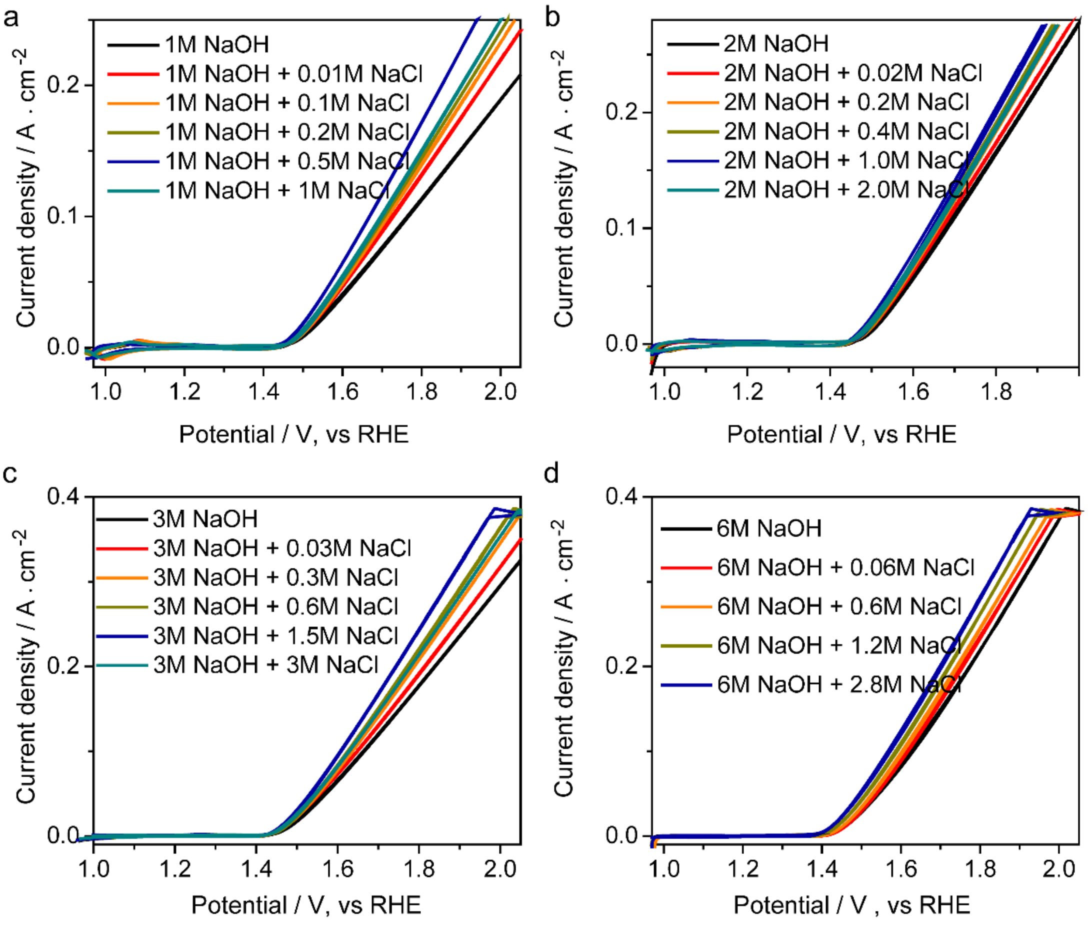

Supplementary Fig. 23 CV curves of Ir/CoFe-LDH recorded in (a) 1 M NaOH, (b) 2 M NaOH, (c) 3 M NaOH, and (d) 6 M NaOH with different concentrations of NaCl.

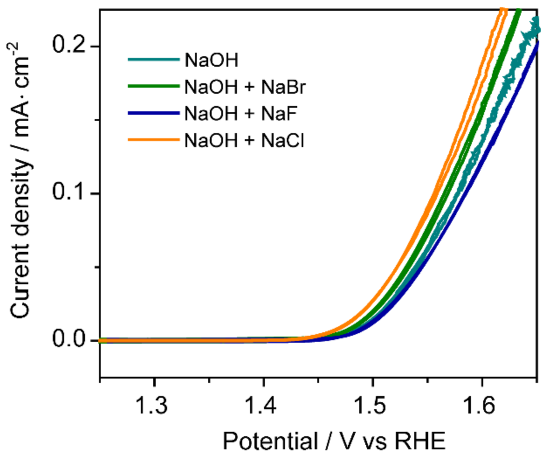

Supplementary Fig.24 The OER performance of Ir/CoFe-LDH in NaOH, NaOH + NaBr, NaOH + NaF, and NaOH + NaCl electrolyte.

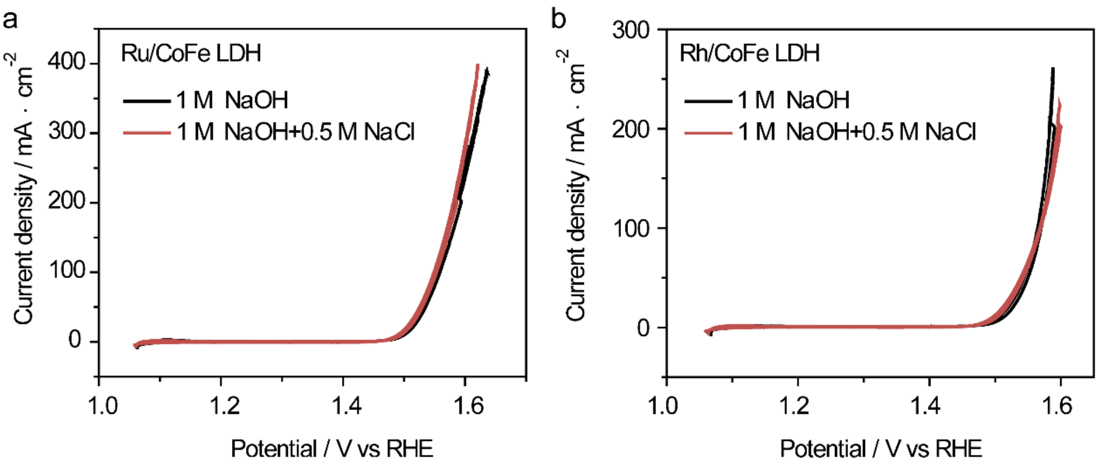

Supplementary Fig. 25 CV curves of (a) Ru/CoFe-LDH and (b) Rh/CoFe-LDH recorded in 1 M NaOH and 1 M NaOH + 0.5 M NaCl electrolyte. The overlapping curves indicated the absence of Cl-adsorption effects.

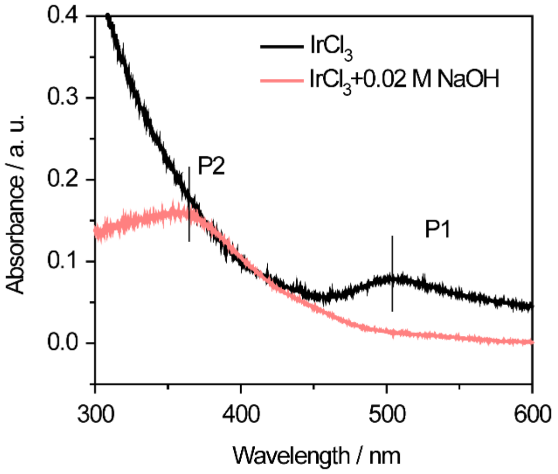

Supplementary Fig. 26 Comparison of UV-vis spectrum of $IrCl_3$ before and after reacting with NaOH.

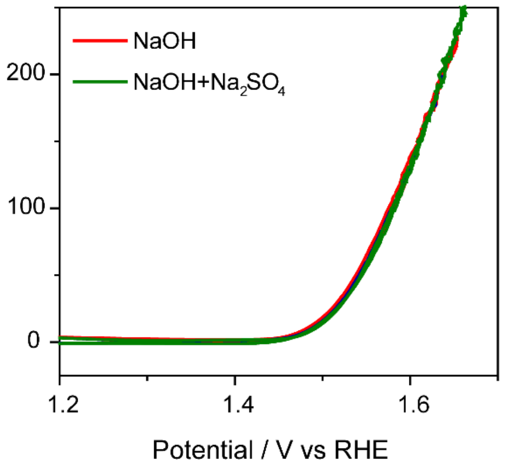

Supplementary Fig.27 The OER performance of Ir/CoFe-LDH in 1 M NaOH and 1 M NaOH + 0.5 M Na_2SO_4.

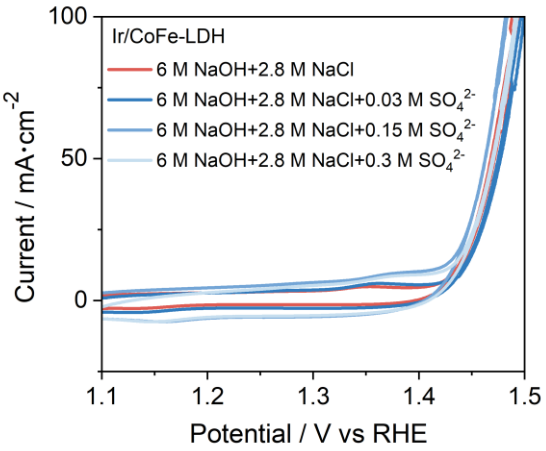

Supplementary Fig.28 CV curves of Ir/CoFe-LDH recorded in 6 M NaOH + 2.8 M NaCl with different SO_4^{2-} concentration (from 0.03 M, representing the sulfate concentration in seawater, to a tenfold enrichment of sulfate concentration, reaching 0.3 M).

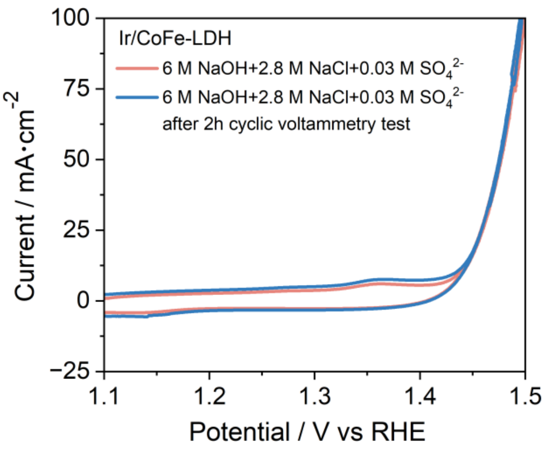

Supplementary Fig.29 The CV curves of Ir/CoFe-LDH before and after a 2-hour cyclic voltammetry test in 6 M NaOH + 2.8 M NaCl + 0.03 M SO_4^{2-}.

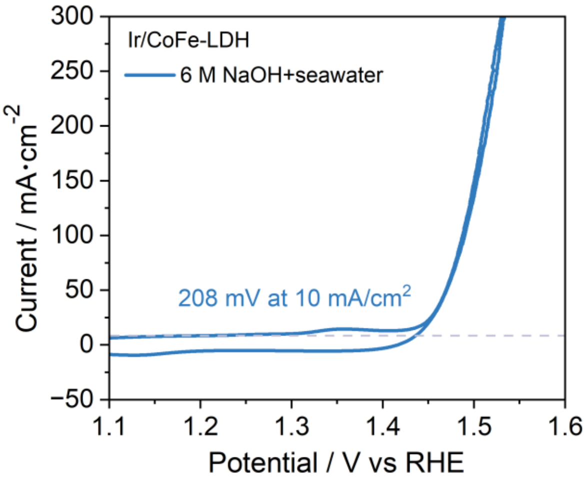

Supplementary Fig.30 The CV curve of Ir/CoFe-LDH in 6 M NaOH + real seawater (calcium and magnesium ions are removed through pre-treatment).

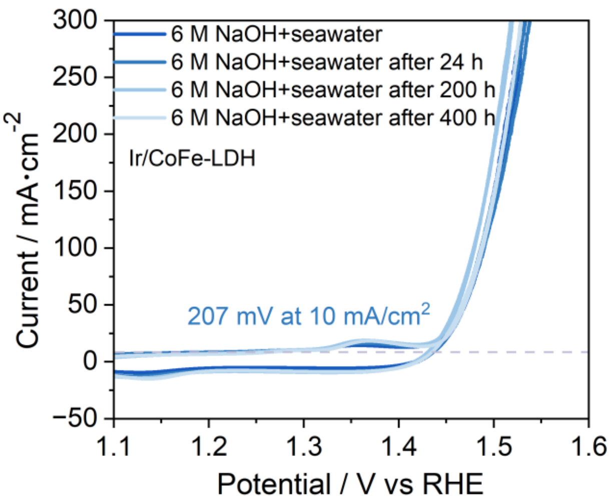

Supplementary Fig.31 The CV curves of Ir/CoFe-LDH before and after long-term stability test in 6 M NaOH + real seawater (calcium and magnesium ions are removed through pre-treatment).

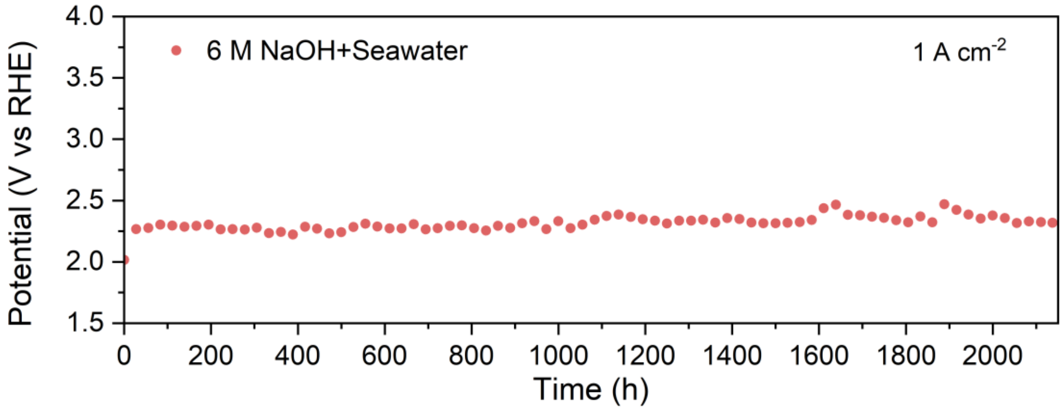

Supplementary Fig.32 The stability test of the Ir/CoFe-LDH//NiCoFeP electrolyzer in real seawater (calcium and magnesium ions are removed through pre-treatment) with 6 M NaOH at a constant current density of 1 A/cm^2.

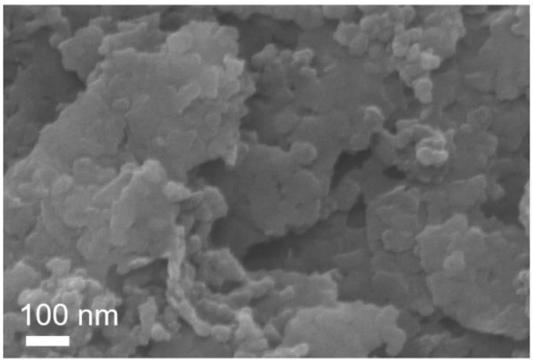

s

Supplementary Fig.33 The SEM image of Ir/CoFe-LDH after long-term stability test in real seawater.

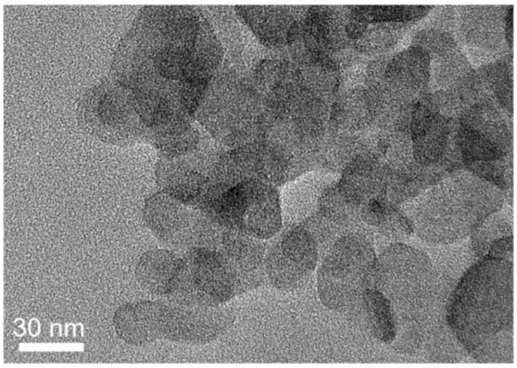

Supplementary Fig.34 The TEM image of Ir/CoFe-LDH after long-term stability test in real seawater.

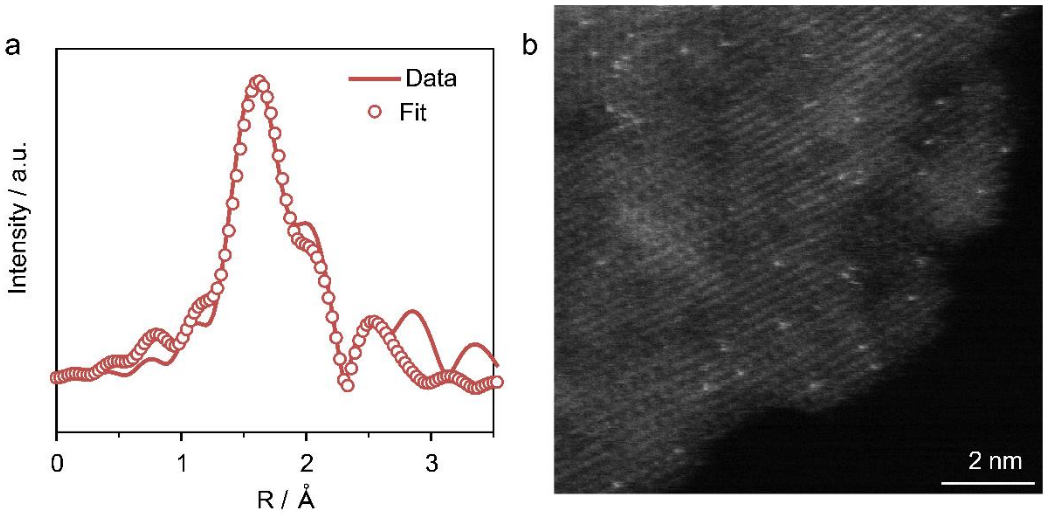

Supplementary Fig. 35 (a) Fourier-transformed Ir L_3-edge EXAFS spectrum and (b) HAADF-STEM image of Ir/CoFe-LDH after long-term stability test.

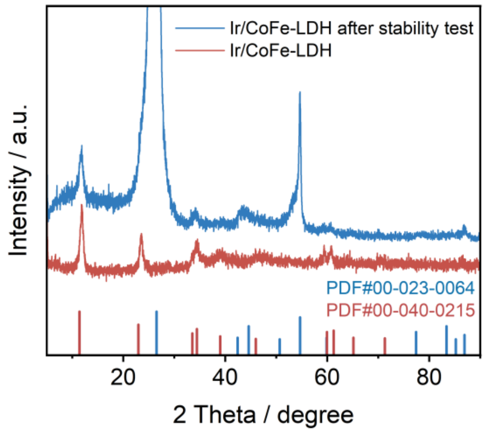

Supplementary Fig. 36 The XRD patterns of Ir/CoFe-LDH after long-term stability test in real seawater.

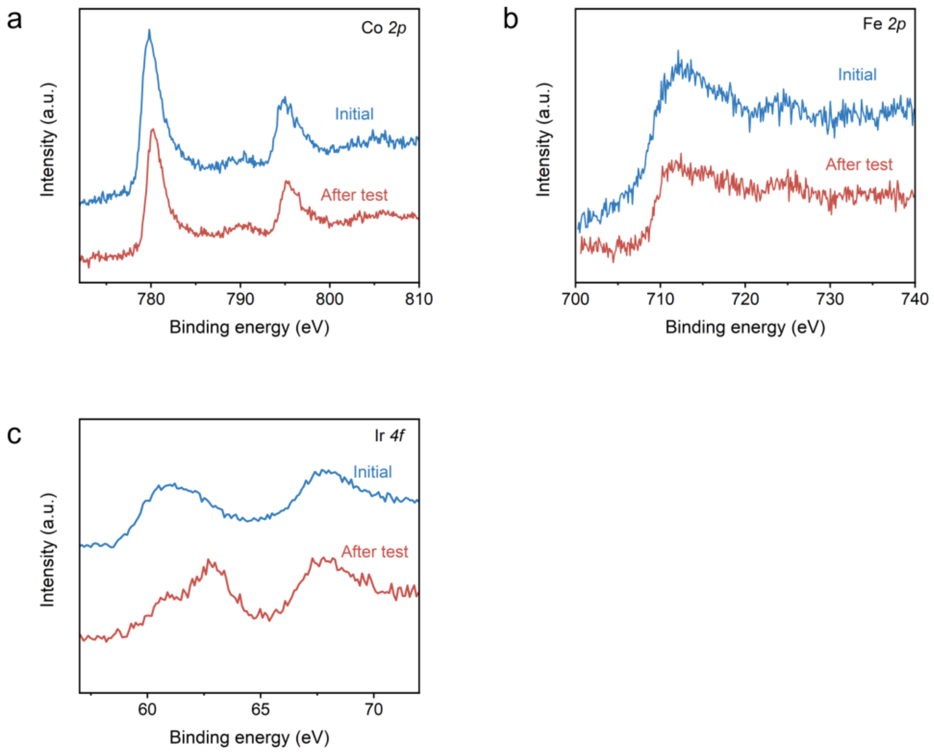

Supplementary Fig. 37 High-resolution XPS spectra of Ir/CoFe-LDH before and after long-term stability test in real seawater. (a) Co 2*p*, (b) Fe 2*p*, and (c) Ir 4*f*.

Supplementary Table 4 Comparison of dissolved Ir, Co, and Fe in electrolyte after long-term OER stability test of Ir/CoFe-LDH and physical mixture of IrO_2 and CoFe-LDH with the same content of Ir.

Catalyst	Ir (ppb)
Ir/CoFe-LDH	9.558
IrO_2/CoFe-LDH	82.308

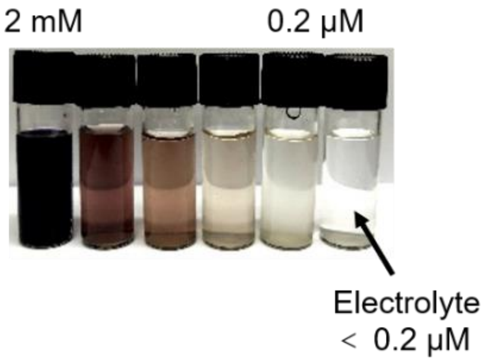

Supplementary Fig. 38 No color reaction between electrolyte (after 1,000 h at 800 mA/cm^2) and starch-KI.

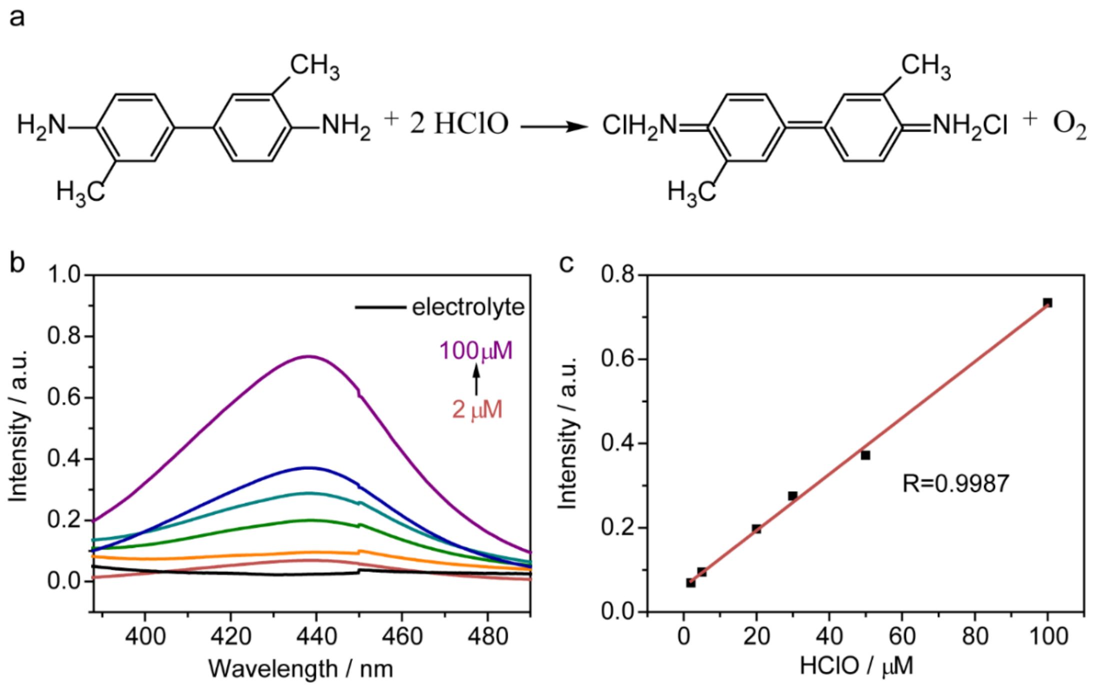

Supplementary Fig. 39 O-tolidine test under constant current condition. (a) The chemical reaction between o-tolidine and HClO. (b) UV-vis spectra of the testing solutions after addition of different amounts of HClO from 2 μM to 100 μM. The black line was the spectra of the testing solutions with electrolyte after OER process, showing

no ClOR. (c) Calibration curve obtained by plotting the concentration of free chlorine against the corresponding absorption peak intensity at 438 nm.

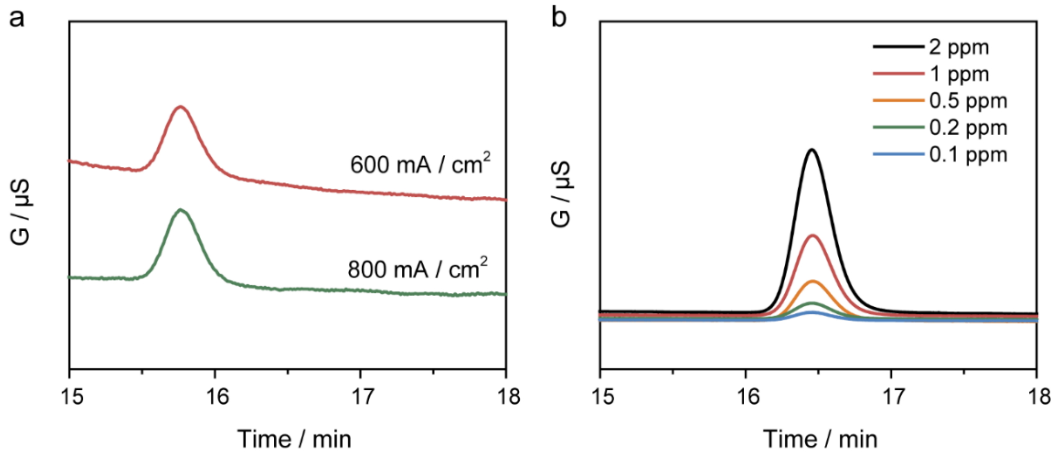

Supplementary Fig. 40 (a) Detection of ClO^- generated over Ir/CoFe-LDH during extended seawater electrolysis using ion chromatography (IC) and (b) IC spectra of HClO solution with different concentrations.

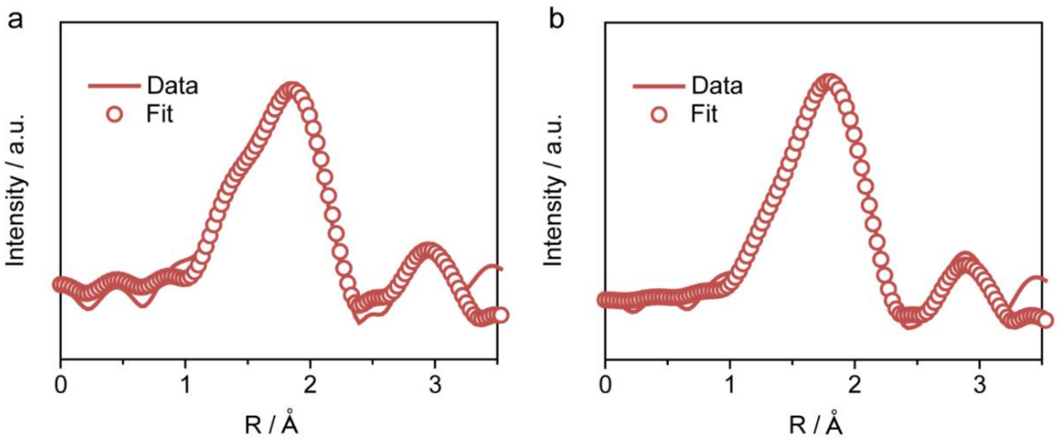

Supplementary Fig. 41 The Fourier-transformed EXAFS spectra of Ir/CoFe-LDH before OER test in (a) NaOH + NaCl and (b) NaOH.

Supplementary Table 5 Fitting data of EXAFS spectrum for Ir/CoFe-LDH before OER.

Sample	Shell	CN	R (Å)	$\Delta\sigma^2 \times 10^3$ ($Å^2$)	ΔE_0 (eV)
NaOH + NaCl	Ir-O	3.1	1.99	0.006	5.50
	Ir-Cl	2.5	2.33	0.004	9.66
	Ir-(O)-Co	2.0	3.20	0.005	12.50
NaOH	Ir-O	3.0	2.00	0.003	5.56
	Ir-Cl	2.8	2.32	0.018	9.34
	Ir-(O)-Co	1.8	3.15	0.005	8.38

CN: coordination number, R: distance between absorber and backscatterer atoms, $\Delta\sigma^2$: disorder term, ΔE_0: inner potential correction. Error bars are estimated to be CN: ± 15%, R: ± 0.02 Å, $\Delta\sigma^2$: ± 20%, and ΔE_0: ± 20%.

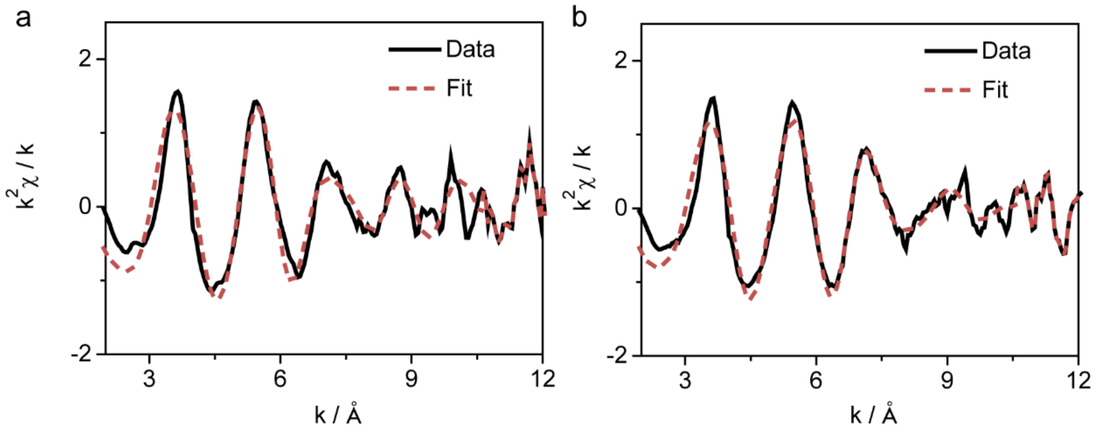

Supplementary Fig. 42 The k space of EXAFS spectrum fitting for Ir/CoFe-LDH recorded at OCV in (a) 1 M NaOH + 0.5 M NaCl and (b) 1 M NaOH.

Supplementary Table 6 Fitting data of EXAFS spectrum for Ir/CoFe-LDH recorded at open circuit voltage.

Sample	Shell	CN	R (Å)	$\Delta\sigma^2\times10^3$ ($Å^2$)	ΔE_0 (eV)
NaOH + NaCl	Ir-O	3.2	1.97	0.006	8.37
	Ir-Cl	2.5	2.31	0.004	7.45
	Ir-(O)-Co	2.0	3.22	0.009	10.0
NaOH	Ir-O	2.9	2.01	0.004	5.97
	Ir-Cl	2.8	2.31	0.008	9.04
	Ir-(O)-Co	2.0	3.10	0.011	10.0

CN: coordination number, *R*: distance between absorber and backscatterer atoms, $\Delta\sigma^2$: disorder term, ΔE_0: inner potential correction. Error bars are estimated to be CN: ± 15%, *R*: ± 0.02 Å, $\Delta\sigma^2$: ± 20%, and ΔE_0: ± 20%.

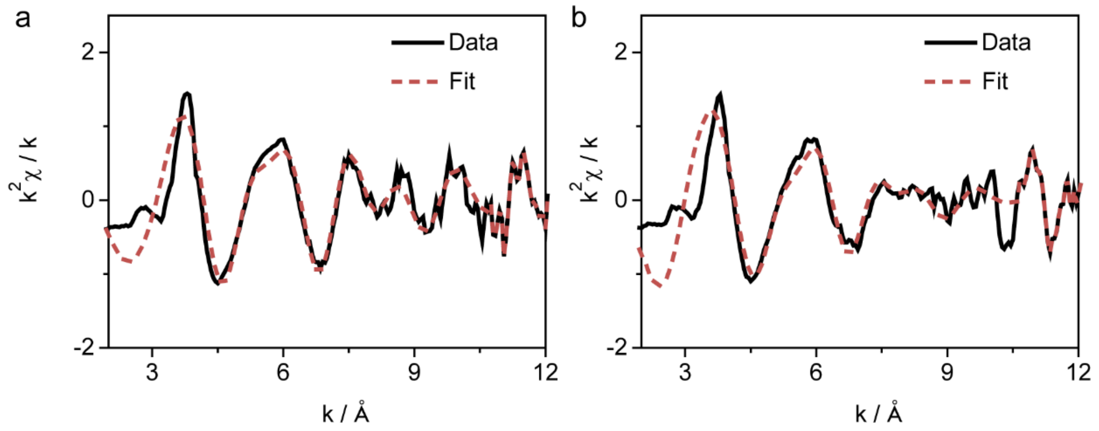

Supplementary Fig. 43 The k space of EXAFS spectrum fitting for Ir/CoFe-LDH recorded at 1.57 V vs RHE in (a) 1 M NaOH + 0.5 M NaCl and (b) 1 M NaOH.

Supplementary Table 7 Fitting data of EXAFS spectrum for Ir/CoFe-LDH recorded at 1.57 V vs RHE.

Sample	Shell	CN	R (Å)	$\Delta\sigma^2 \times 10^3$ ($Å^2$)	ΔE_0 (eV)
NaOH + NaCl	Ir-O	4.0	1.91	0.003	5.94
	Ir-Cl	1.2	2.28	0.003	12.09
	Ir-(O)-Co	2.7	2.93	0.008	12.34
NaOH	Ir-O	5.9	1.94	0.011	5.903
	Ir-(O)-Co	3.5	3.02	0.015	17.51

CN: coordination number, *R*: distance between absorber and backscatterer atoms, $\Delta\sigma^2$: disorder term, ΔE_0: inner potential correction. Error bars are estimated to be CN: ± 15%, *R*: ± 0.02 Å, $\Delta\sigma^2$: ± 20%, and ΔE_0: ± 20%.

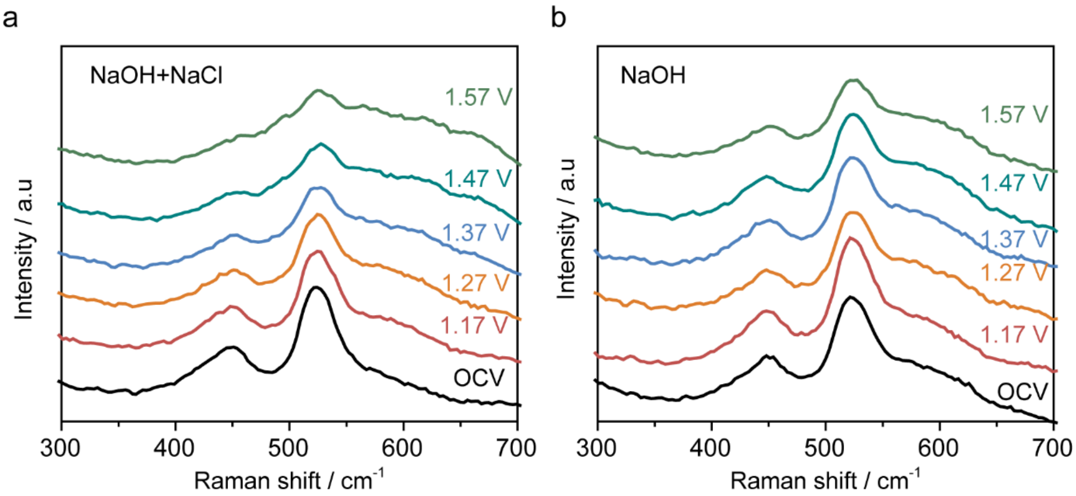

Supplementary Fig. 44 *In situ* Raman spectra of CoFe-LDH recorded in (a) NaOH + NaCl and (b) NaOH.

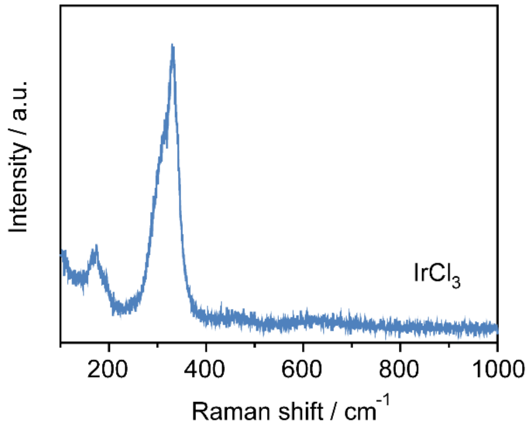

Supplementary Fig. 45 Raman spectra of $IrCl_3$ standard sample.

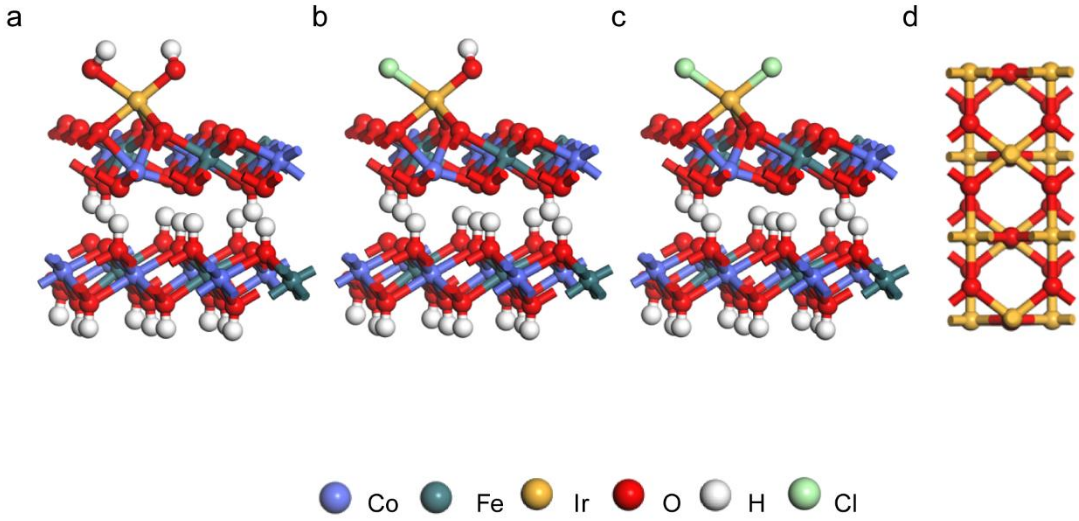

Supplementary Fig. 46 Optimized structure of (a) $Ir_{-OH,OH}$, (b) $Ir_{-Cl,OH}$, (c) $Ir_{-Cl,Cl}$ and (d) IrO_2 (yellow: Ir, dark yellow: Fe, grey: Co, red: O, white: H, green: Cl).

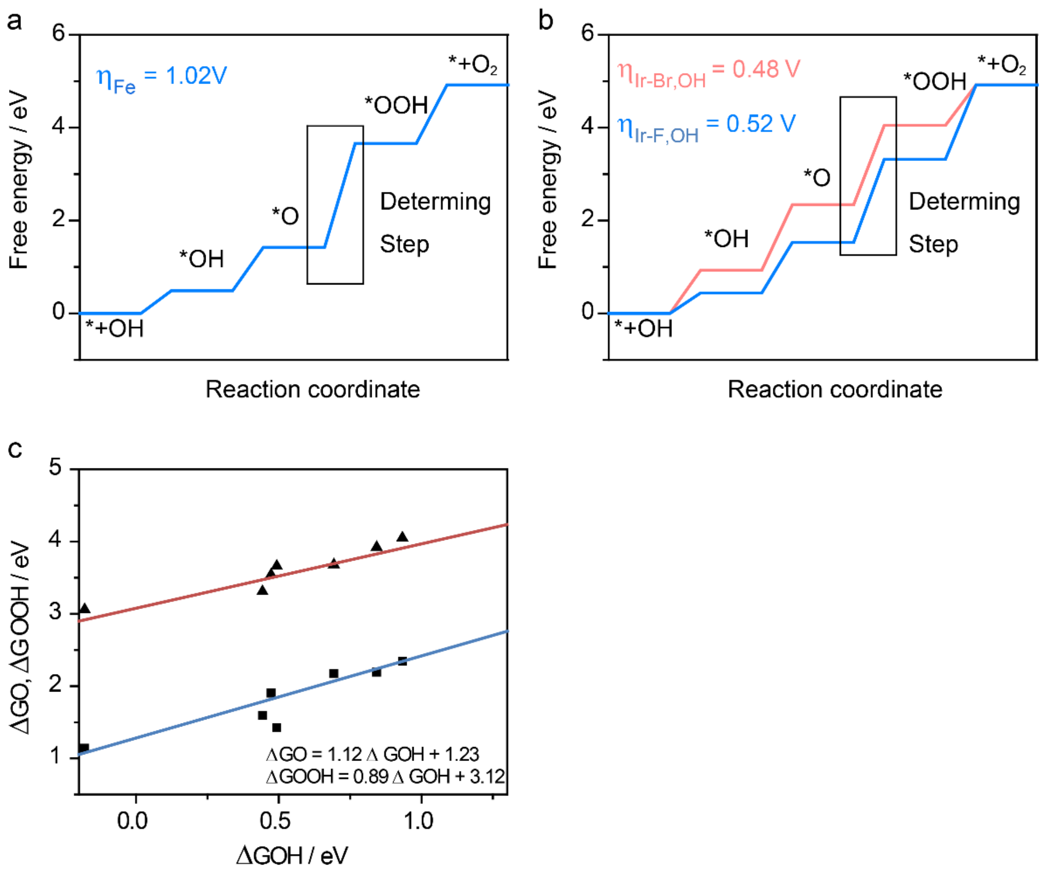

Supplementary Fig. 47 Theoretical OER free energy of (a) CoFe-LDH and (b) $Ir_{-OH,F}$ and $Ir_{-OH,Br}$. (c) Scaling relations between △G_{OOH} (red), △G_{O} (blue) and △G_{OH}.

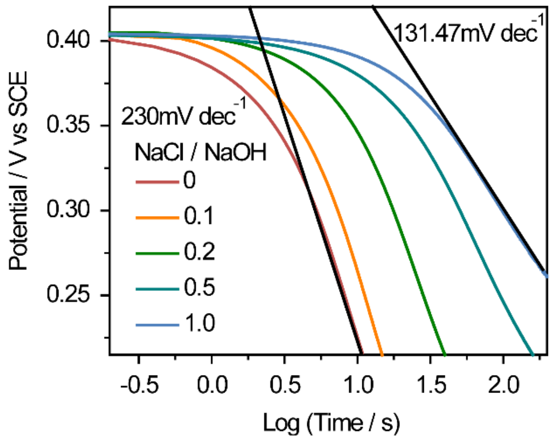

Supplementary Fig. 48 OCP decay as a function of time after 4 min of polarization at an overpotential of 200 mV.

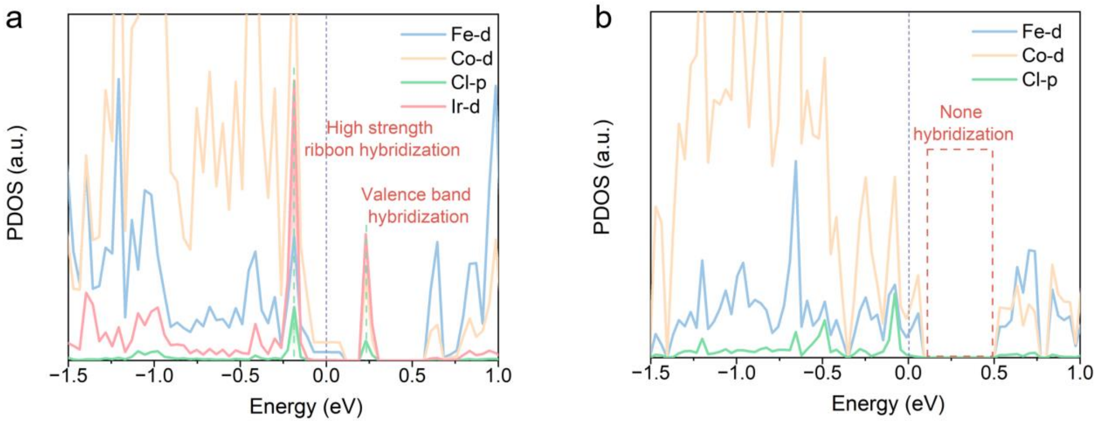

Supplementary Fig. 49 PDOS of (a) Cl with Ir/CoFe-LDH and (b) Cl with CoFe-LDH.

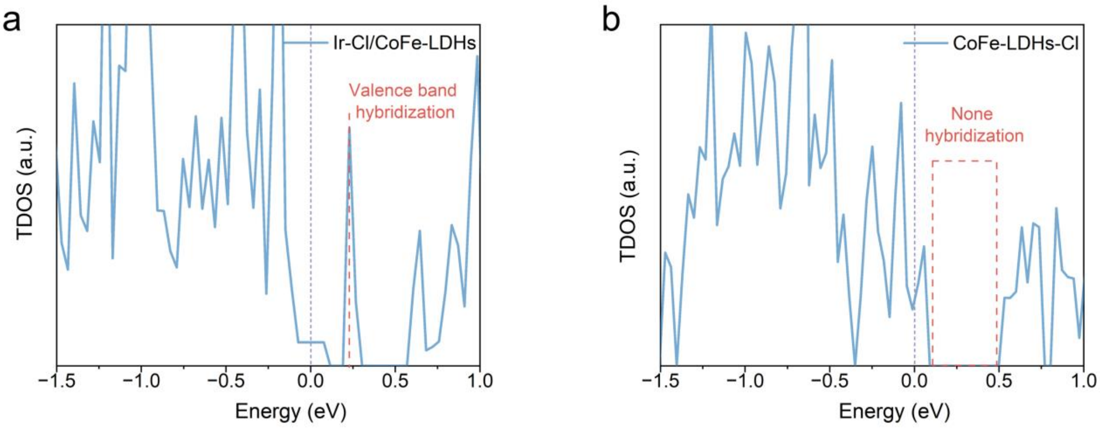

Supplementary Fig. 50 TDOS of (a) Cl with Ir/CoFe-LDH and (b) Cl with CoFe-LDH.

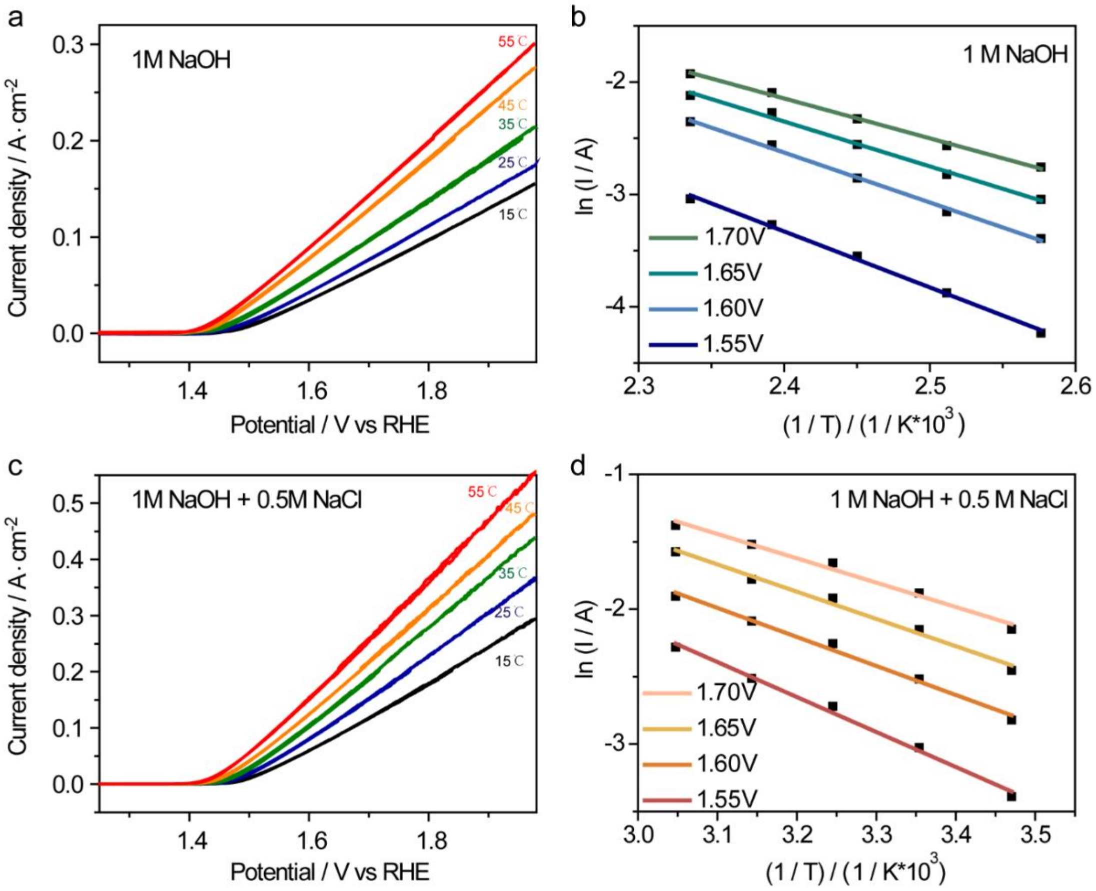

Supplementary Fig. 51 OER polarization curves measured at different temperatures (15-55°C) without IR correction, and the Arrhenius plots in 1 M NaOH (a & b) and 1 M NaOH + 0.5 M NaCl (c & d).

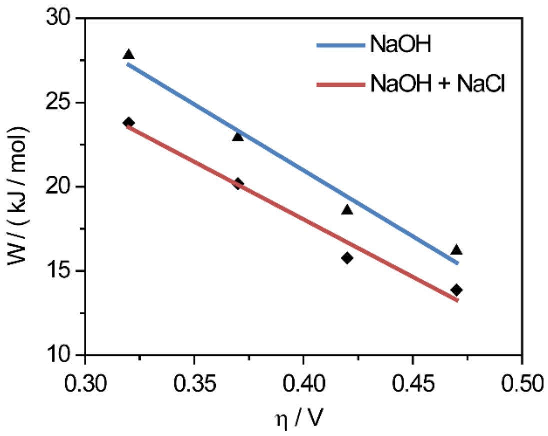

Supplementary Fig. 52 Activation energy of Ir/CoFe-LDH in 1 M NaOH and 1 M NaOH + 0.5 M NaCl.

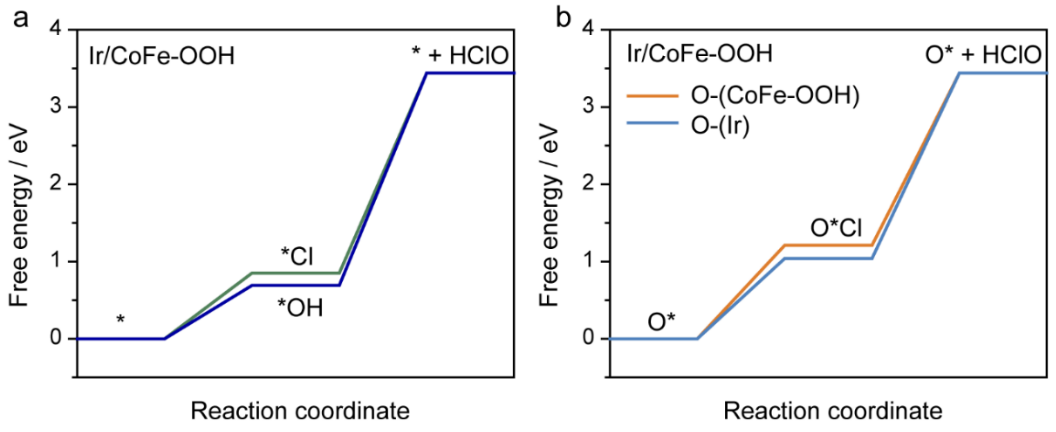

Supplementary Fig. 53 The free energy diagram on (a) Ir sites and (b) surface O atoms.

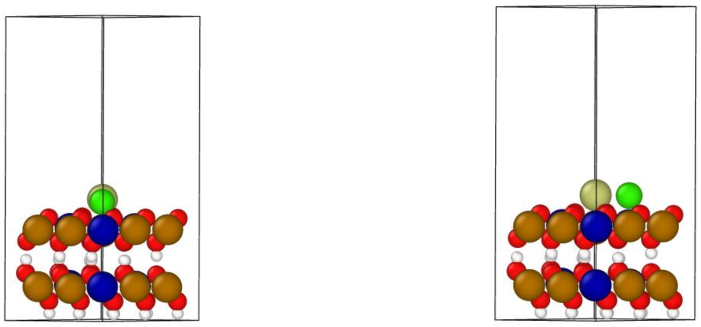

Supplementary Fig. 54 The Cl movement trajectory movie (provided in attachment files) during the formation of stable configurations: from (left) Co-Cl to Ir-Cl, and from (right) Fe-Cl to Ir-Cl. (yellow: Fe, blue: Co, grey: Ir, green: Cl, red: O, pink: H).

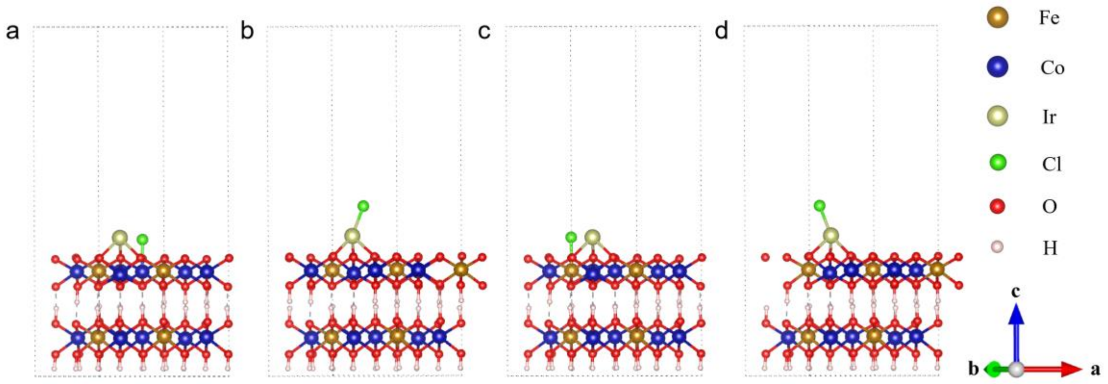

Supplementary Fig. 55 Structure evolution during structural optimization/relaxation: from (a) Co-Cl to (b) Ir-Cl, and from (c) Fe-Cl to (d) Ir-Cl (yellow: Fe, blue: Co, grey: Ir, green: Cl, red: O, pink: H).

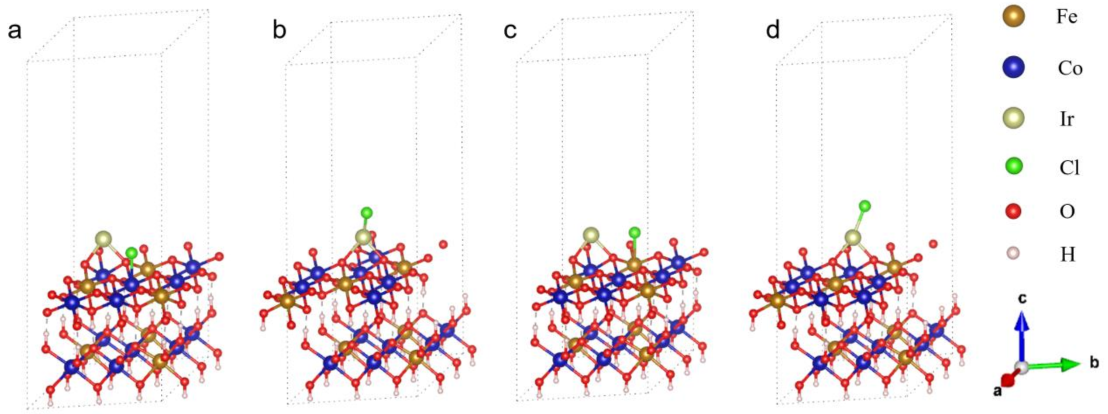

Supplementary Fig. 56 Structure evolution during structural optimization/relaxation: from (a) Co-Cl to (b) Ir-Cl, and from (c) Fe-Cl to (d) Ir-Cl (yellow: Fe, blue: Co, grey: Ir, green: Cl, red: O, pink: H).

References

1. Li, P. et al. Common-ion effect triggered highly sustained seawater electrolysis with additional NaCl production. *Research* 2872141 (2020).
