# Supplementary material for: Dynamic chloride ion adsorption on single iridium atom boosts seawater oxidation catalysis
Source: Nat Commun. 2024 Mar 4;15:1973. doi: 10.1038/s41467-024-46140-y (PMC10912682; doi:10.1038/s41467-024-46140-y)
Supplement: Supplementary file 3 — Description of Additional Supplementary Files [file 41467_2024_46140_MOESM3_ESM.pdf]

# Description of Additional Supplementary Files

**Supplementary Movie Legends**

**Supplementary Movie 1:** From Co-Cl to Ir-Cl_The Cl movement trajectory GIF during the formation of stable configurations

**Supplementary Movie 2:** From Fe-Cl to Ir-Cl_The Cl movement trajectory GIF during the formation of stable configurations
